# Forty Years of Lunacy Work
**Recollections of Forty Years: Being an Account at first hand of some famous criminal lunacy cases, English and American.* By L. Forbes Winslow. M.B., D.C.L., LL.D. London : John Ouseley, Ltd., Fleet Lane, E.C. 12s. 6d. net.


**Published:** 1910-10-01

**Authors:** 


### FORTY YEARS OF LUNACY WORK.*

### DR. FORBES WINSLOW'S RECOLLECTIONS OF TRIALS AND CASES.

Dr. Forbes Winslow needs no introduction to the profession; perhaps just for that reason is it that his recollections will be so interesting to many readers. The earlier part of this book deals largely with personal experiences, and, indeed, the personal note dominates the work. No one can read these trenchant sentences and forcibly expressed opinions without feeling that the man who wrote and who held them is an individual who on occasions has felt it necessary to stand

> Fronting his peers and those below his ken
> And those above, with scorn of consequence.

It needs a great deal of originality, or personality, above all, of assurance that right is right, to be able to do that, and those of us who are unprejudiced and equal to the task of reading without attempting to judge, will find this life history of one who has been so much in the limelight, not only interesting but instructive.

Forbes Winslow comes from a medical family, and saw the light for the first time, as the saying is, in Guildford Street on the last day of January 1844. His father "was a physician, struggling to establish the recognition of the plea of insanity in criminal cases in England, and he lived to see the accomplishment of his desire." We have glimpses in these pages of Dr. Winslow, the elder, and they show us a lovable but strong-minded personality, from whom doubtless his son has inherited some part of the persevering determination that is so evident in his career. These early recollections, and personal experiences are in some ways slight: they are minutiæ, peeps into the porridge pot, attempts by the fisher himself to tell the history of the first fishing of the murex, but all the same they are vastly entertaining, and here and there they furnish a remark or an anecdote which is of historical interest. The book is a sensational one, though not in the ordinary sense of the term. Its sensationalism lingers: it does not evaporate with the morbid interest of a murder case or the ephemeral life of a "horror" or a "mystery." Those who delight in the reading of autobiographies will find it a readable and an interesting book, and those who wish to have something more than autobiography will find it pregnant with material for thoughtful consideration.

#### A Score of Murder Cases.

In these pages are told—interestingly, for the author can narrate with a skill that pins attention to the end—the story of many a famous (or shall we say notorious?) trial to which reference is made in ordinary text-books on medical jurisprudence. Here we have, for instance, details of the following cases: Nunn *v.* Hemming, Robert King, the "Old Kent Road murder," the Bravo mystery, the Penge case, the Dodwell case, the Mainwaring case, the Brighton Line murder, Mrs. Maybrick, Mrs. Pearcey, Drent and Treadaway, the Reading child-farming case, Mary Ansell's case, the Trunk Murder, the case of Eliza Leitner, the case of Margaret Lemon, the Clifton Lunacy case, the case of Dr. Story, tried for arson, the Townshend Lunacy Commission, Jack the Ripper, Hannigan, Durrant, the American church murder, the case of Mrs. Fleming, and many others. Several of these are well-known murder cases, and it is worthy of consideration that Dr. Winslow thinks that in nearly every one of these in which the jury found the prisoner guilty there was strong evidence to show that the murderer was insane at the time the crime had been committed. In some, the public will remember that the efforts for mitigation of sentence, in several successful, were initiated by him, and it is satisfactory to find that in these attempts—even in the Maybrick case, where the leading medical organ at first opposed a commutation—the profession manfully supported him. As a humanitarian Dr. Winslow's record is a brilliant one, and although we confess that we do not always agree with him in his arguments, we are sufficiently conscious of the fact that his vast experience in, and close study of, the subjects with which he deals enable him to speak as an authority whose views should not be treated lightly even in these very debatable matters.

#### Jack the Ripper.

Naturally, in view of the recent recrudescence of attention to the notorious Whitechapel murders, his chapter on Jack the Ripper will appeal very strongly to those who take an interest in that unsolved mystery. It is an absorbing account of the reign of terror that the East End crimes and mutilations brought into being, and no less enthralling is the author's story of his "reconstruction" of the murders. He holds that the murderer was a man who suffered from religious monomania, with lucid intervals, during which he was in every way unconscious of what had taken place. Patiently following each clue and with the detective instinct

* *Recollections of Forty Years: Being an Account at first hand of some famous criminal lunacy cases, English and American.* By L. Forbes Winslow, M.B., D.C.L., LL.D. London: John Ouseley, Ltd., Fleet Lane, E.C. 12s. 6d. net.

that is after all only another name for sound logical reasoning, Dr. Winslow established a more or less conclusive case against a certain individual, who now, as he thinks, is in South Africa, but the police who were fully acquainted with the evidence collected by the author, refused to interfere. For the full history of these remarkable tragedies we must refer to Dr. Winslow's book, which teems with interesting and instructive points on the case hitherto unpublished.

The trunk murder of 1905 will yet be fresh in the recollection of many. The accused, Devereux, was put on trial for having murdered his wife and twin children by poison and then secreting their bodies in a trunk which was consigned to a luggage depository. The case aroused a large amount of excitement, and feeling against the prisoner, from the first, reached a very high point. At the trial public opinion was prejudiced against him, and reading the record five years later, in the light of the revelations made by Dr. Winslow, one is oppressed by a serious doubt whether or not Devereux had a fair trial. His own statement, from which he never budged, was that he had found his wife and children dead in their beds, the room smelling strongly of chloroform, and that, in a moment of panic he had lost his head and concealed the bodies in a trunk. The man's family history was at any rate strongly suggestive of a hereditary taint of insanity. His grandfather committed suicide, his father attempted to commit suicide by hanging; on his maternal side the history is even more suggestive. He himself was neurotic, irritable, and quarrelsome, and when on his trial he displayed signs of mental instability. In prison he was exalted, incoherent, and eccentric, but Dr. Maudsley, who examined him, thought that his insanity was simulated and that his actions showed no signs of mental aberration. Dr. Winslow, who examined him shortly afterwards, and whose report is now published *in extenso*, is of opinion that Devereux was certainly insane: the list of objective symptoms given, apart altogether from the record of action, conduct and speech, is strongly supportive of this verdict. Nevertheless, though Dr. Talbot MacCarthy agreed with Dr. Winslow's opinion, the plea of insanity was not accepted, and Devereux was sentenced to death and ultimately executed, all attempts to procure a reprieve proving abortive. Dr. Winslow suggests that he was entirely innocent of the crime, and accepts the prisoner's explanation in full. In the circumstances one can only say that the author appears to have reasonable grounds for holding that view.

### The Train Murder and the Reading Case.

Similarly in Mrs. Dyer's and in Lefroy's case, Dr. Winslow thinks that the prisoners should not have been executed, since they were evidently insane. A more flagrant instance of "treating insanity by hanging" than that of Mary Ansell, hanged for the murder of her sister, it would be hard to find. This woman, as the author shows, was a weak-minded, irresponsible person; the agitation for a reprieve in her case was strongly supported and yet it was ineffectual. All the other cases, however much they differ in detail from these four already cited, give food for reflection as to the justice of our present system under which imbeciles and irresponsibles are treated like ordinary persons unless they can prove to the satisfaction of a judge, who may be entirely ignorant of psychiatry, and a jury not only ignorant but prejudiced, that they are madmen or madwomen in the boisterous sense that appeals to the public.

It is just this reflection on our juridical methods that makes Dr. Winslow's book so interesting to thoughtful readers. How is a person accused of murder, with a mass of popular indignation thrown against him, with a prejudiced press, that knows nothing, or next to nothing, of psychiatry, opposing him and calumniating his defenders—how is such an unfortunate to win in a struggle in which loss means the gallows, and victory after all only Broadmoor? Very recent happenings have given us a melancholy comment on this question, and all these cases here enumerated are still more definite proofs of the fact that there is something rotten in the system that prevails with us. In Germany they are at any rate more scientific, if not more humane. Only this week an unfortunate man, who was obviously insane, was executed in France under singularly revolting circumstances; but readers will doubtless remember the notorious Blecher case in Berlin, a year or two ago, in which the expert evidence proved that the murderer was irresponsible, and a just sentence was passed, notwithstanding the fiercest public indignation and excitement. The details of the case are too fresh in the memory to be here recorded, but it may be pointed out that the police lost no time in communicating to the Press the official findings of the experts, and that this communication did much to allay the tremendous prejudice against the "monster" who had cut up a child. With us the policy appears to be singularly destitute of common-sense. Expert witnesses for the defence are bullied, their motives called into question, and their attitude distorted, simply because they dare to affirm that a murderer or a great criminal is in the majority of cases an ill man who is not responsible for his actions. The matter of legal responsibility is now sufficiently thrashed out, but this other matter of expert witnesses is still, as Dr. Winslow shows, in need of reform. An expert witness is not a partisan: he gives an opinion according to his experience and knowledge, and if he happens to differ from the Crown experts, however capable these latter are, it is monstrous to accuse him of perjury committed to earn a fee which, as Dr. Winslow again shows, is more often than not never paid. The whole system of our trial by jury, in the light of the experiences, appears to be by no means so sacrosanct as some of us imagine. This is not an old heresy—but it is worth repeating until at least the public understands that a heresy is sometimes a truth.

We can only touch upon Dr. Winslow's book in parts. The whole remains for the reader who buys it and peruses it carefully. It is well worth buying and perusing, for it is not merely a *livre d'occasion*, but a work that has a permanent benefit as a contribution to the medical history of our times.

